# Treatment of Syphilitic Buboes

**Published:** 1876-08

**Authors:** 


Treatment of Syphilitic Buboes by Parenchymatous Injection of Iodide of Potassium.—Dr. Jacubowitz, of Nagy-Karoly (*Der Praktische Arzt*, No. 7, Jahrgang xvi.), starts from the principle that no inflammation to which a degenerative action is attributable is occasioned by the injections, but by this means a solvent of a non-irritating character is brought into direct contact with the glandular tissue. He avoids tincture of iodine, all alcoholic fluids and carbolic acid, and uses instead a weak solution of iodide of potassium in the proportion of about one part to thirty of water. He gives two cases in which he obtained extraordinarily successful results. In one case he made a puncture into the most prominent part of a gland which was enlarged to the size of a goose's egg, pushing the needle in obliquely to a considerable distance. After injecting about the fourth of the syringeful a resistance was felt; he then withdrew the needle for a short distance, penetrated a septum on one side, and again injected a quarter part. By repeating this process, he threw in about fifteen grains of the iodide in an ounce of water. The tumor almost immediately became harder, smaller, and less painful. After four injections performed in the course of two days the tumor gradually dwindled to the size of a hazelnut, and ultimately vanished altogether. The second case was very similar. Here, however, two dark-blue bodies remained, which were so hard that it seemed to be impossible to inject them. Dr. Jacubowitz, however, injected hypedermically the iodide on two occasions with perfect success. Ten injections were required altogether. The small quantity of the iodide required to produce the effects observed is very remarkable.—*Practitioner*, June, 1876.

Eucalyptus in Ague.—Dr. Paul Boyce says, in the Vriginia *Medical Monthly:* A case of ague and fever, which had been treated with quinia, arsenic, etc., was cured in four days by simply taking the essence of eucalyptus (prepared from the oil distilled from the leaves) ʒij three times a day. I believe we possess in the eucalyptus a remedy not inferior to the cinchona alkaloids. The oil applied to the nerve of a tooth soon destroys its sensitiveness and quiets the pain. In purulent catarrhal affections of the urethra it acts like a charm.—*Drug. Cir. and Chem. Gazette.*

